# Effect of Colchicine Use and Usual Care Alone on the Rate of Progression to Chronic Kidney Disease and Mortality in Patients with Heat-Related Injury

**DOI:** 10.3390/healthcare14060744

**Published:** 2026-03-16

**Authors:** Min-Feng Tseng, Chi-Hsiang Chung, Wu-Chien Chien, Shang-Jyh Hwang, Yi-Shiou Chiou, Chia-Chao Wu

**Affiliations:** 1 Doctoral Degree Program in Toxicology, College of Pharmacy, Kaohsiung Medical University, Kaohsiung 807378, Taiwan; a0978060006@mail.ngh.com.tw; 2 Department of Internal Medicine, Zuoying Armed Forces General Hospital, Kaohsiung 813204, Taiwan; 3 Division of Nephrology, Department of Internal Medicine, Tri-Service General Hospital, National Defense Medical University, Taipei 114201, Taiwan; 4 Department of Medical Research, Tri-Service General Hospital, National Defense Medical University, Taipei 114201, Taiwan; g694810042@mail.ndmctsgh.edu.tw (C.-H.C.); chienwu@mail.ndmctsgh.edu.tw (W.-C.C.); 5 School of Public Health, College of Public Health, National Defense Medical University, Taipei 114201, Taiwan; 6 Division of Nephrology, Department of Internal Medicine, Kaohsiung Medical University Hospital, Kaohsiung Medical University, Kaohsiung 807378, Taiwan; sjhwang@kmu.edu.tw; 7 Faculty of Medicine, College of Medicine, Kaohsiung Medical University, Kaohsiung 807378, Taiwan; 8 Master/Doctoral Degree Program in Toxicology, College of Pharmacy, Kaohsiung Medical University, Kaohsiung 807378, Taiwan

**Keywords:** heat-related injury, chronic kidney disease, colchicine, inflammation, renal outcomes, nationwide cohort study

## Abstract

**Highlights:**

**What are the main findings?**

- Colchicine use after heat-related injury was associated with a reduced long-term risk of CKD progression and dialysis initiation.
- No significant association was observed between colchicine use and all-cause mortality in HRI patients.

**What are the implications of the main findings?**

- Anti-inflammatory therapy may represent a potential strategy for mitigating long-term renal sequelae following heat-related injury.
- These findings support further prospective studies to clarify the role of colchicine in post-HRI renal protection.

**Abstract:**

Background: Heat-related injury (HRI) induces systemic inflammation and is associated with acute kidney injury and subsequent progression to chronic kidney disease (CKD). Currently, no established pharmacological intervention exists to prevent long-term renal deterioration following HRI. This study aimed to evaluate the association between colchicine use and long-term renal outcomes in patients with HRI. Methods: We conducted a nationwide retrospective cohort study using data from the Taiwanese National Health Insurance Research Database. Adult patients diagnosed with HRI between 2000 and 2015 were identified. Colchicine users were defined as patients who received colchicine within 7 days after the index HRI event and were compared with propensity score-matched nonusers. The primary outcomes included CKD progression, initiation of hemodialysis, CKD-related mortality, and all-cause mortality. Results: A total of 4961 eligible patients with HRI were included in the analysis. During a median follow-up of 9.65 years, colchicine use was associated with a significantly lower risk of CKD progression, initiation of hemodialysis, and CKD-related mortality compared with nonuse. No significant association was observed between colchicine use and all-cause mortality. Conclusions: Colchicine use following HRI was associated with favorable long-term renal outcomes, including reduced risks of CKD progression and dialysis. Although causal inference cannot be established due to the observational design, these findings suggest a potential renoprotective association of colchicine in patients with HRI.

## 1. Introduction

Globally, the increase in temperature has contributed to a markedly increased risk of morbidity and mortality. The intense summer heat waves experienced worldwide over the past decades have led to an increased risk of heat-related illness (HRI). The Center for Disease Control and Prevention (CDC) reported that during 2004–2018, an average of 702 heat-related deaths occurred in the United States annually. Natural heat exposure can contribute to death due to chronic medical disease, alcohol poisoning, or drug overdoses [1].

Chronic kidney disease (CKD) can develop as a consequence of chronic conditions such as diabetes mellitus (DM) and hypertension. Furthermore, CKD is associated with several adverse psychosocial and socioeconomic burdens on health, including reduced quality of life, poor participation in life, and mental illnesses [2]. Acute kidney injury (AKI) due to heat stroke is reportedly associated with the development of CKD. Recurrent heat strokes and dehydration can result in CKD and multiple organ damage in animals and humans. This may play a role in the epidemic of CKD in the hottest regions of the world where workers are exposed to extreme heat [3,4,5,6,7].

Elevated body temperatures can lead to an increase in core temperatures, blood hyperosmolality, and dehydration. Because heat accumulation exceeds heat dissipation during exposure to environmental heat stress or heavy exercise, HRIs may progress from heat exhaustion to heatstroke. Heatstroke is a severe HRI that results in a body temperature of >40.0 °C, dizziness, headache and confusion. Accumulating evidence suggests that some patients with heat injury can develop organ dysfunction, death, or excessive inflammation due to the hyperthermia itself and the ischemia caused by reduced blood flow [8]. Both AKI and rhabdomyolysis are the most common types of renal-associated injury. Importantly, rhabdomyolysis contributes to renal injury primarily through myoglobin-induced acute kidney injury rather than acting as an independent cause of chronic kidney disease.

Rhabdomyolysis affects renal function primarily through myoglobin-induced acute kidney injury rather than as an independent renal disorder. Impairment of thermoregulatory mechanisms leads to cellular dysfunction, characterized by exaggerated inflammatory responses and direct cytotoxicity, which may culminate in circulatory collapse and multiorgan failure [9].

Heat stress and dehydration also play a role in kidney stone formation, and poor hydration habits may increase the risk of recurrent urinary tract infections. The resultant social and economic consequences include disability and loss of productivity and employment. Given the rise in temperatures globally, there is a need to better understand how heat stress can induce kidney diseases, determine strategies to provide adequate hydration, identify methods to reduce the negative effects of chronic heat exposure, and explore preventive strategies and the risk factors of HRI.

Colchicine is an inexpensive, orally administered anti-inflammatory agent derived from the autumn crocus and widely used for gout, Behçet’s disease, pericarditis, coronary artery disease, and other inflammatory conditions [10]. Its anti-inflammatory effects involve inhibition of tubulin polymerization, suppression of leukocyte chemotaxis, and modulation of innate immunity, and recent studies suggest potential benefits in reducing inflammation-related complications of COVID-19, including hospitalization and mortality [11,12]. Heat-related injury (HRI) triggers a systemic inflammatory response characterized by endothelial injury, increased intestinal permeability, endotoxin translocation, and elevated pro-inflammatory cytokines such as IL-1β, IL-6, TNF-α, and IL-18 [13], leading to renal hypoperfusion, tubular injury, acute kidney injury, and subsequent risk of chronic kidney disease progression. By inhibiting neutrophil activation and suppressing NLRP3 inflammasome–mediated IL-1β signaling, colchicine provides a biologically plausible strategy to attenuate heat-induced inflammation; however, current HRI management remains limited to cooling, fluid resuscitation, and supportive care, with no established pharmacologic therapy to prevent long-term renal deterioration.

However, studies on the application of colchicine in HRI are lacking. We hypothesized that the anti-inflammatory action and acceptable safety profile of colchicine makes it a safe and effective treatment choice for heat-induced systemic inflammatory response syndrome due to cytokine release. Therefore, in this retrospective cohort study, we aimed to compare long-term renal and mortality outcomes between colchicine users and nonusers among patients with heat-related injury.

## 2. Methods and Materials

### 2.1. Data Sources and Record Linkage

The National Health Insurance Research Database (NHIRD) is a population-based database that records data of beneficiaries of the National Health Insurance (NHI) program in Taiwan, maintained by the National Health Insurance Administration, Ministry of Health and Welfare (Taipei, Taiwan) [14]. Currently, the NHI covers more than 99% of Taiwan’s population, and the NHIRD has been widely used in epidemiological studies evaluating therapeutic interventions and disease outcomes [15,16,17,18].

We extracted the data of individuals diagnosed with HRI from the NHIRD and confirmed the findings using data obtained from cohorts included in the Catastrophic Illness Patient Database, for which histological confirmation of HRI is required for recruitment.

### 2.2. Study Participants

This retrospective population-based cohort study was approved by the Institutional Review Board (IRB) of Kaohsiung Armed Forces General Hospital (No: KAFGHIRB 110-22). Because all data were anonymized before collection, the IRB waived the need for informed consent.

Patients with HRI were identified using the International Classification of Diseases, Ninth Revision (ICD-9) code 992 from 1 January 2000, to 31 December 2015. This retrospective cohort study included patients with heat-related injury (HRI) identified using ICD-9-CM code 992 from the Taiwanese National Health Insurance Research Database. Among this HRI cohort, patients who received colchicine within 7 days after the index HRI event were defined as the exposure group, and those who did not receive colchicine constituted the comparison group. The primary comparison of this study was therefore between colchicine users and nonusers within a cohort of patients with HRI. The authors accessed the NHIRD of Taiwanese to retrieve the data utilized in their analysis on 6 August 2022, which corresponds to the timestamp of the processing syntax at the time of data export. Individuals who were diagnosed with HRI were included in the study. Patients with incomplete data, HRI diagnosed before 2000, CKD or renal replacement therapy, history of kidney surgery, acute renal injury, urinary system infection, anomaly of genital organs, obstructive kidney diseases, glomerulonephritis, or nephritic syndrome, unknown sex, and age < 18 years or >100 years were excluded from the study.

Furthermore, to enhance diagnostic accuracy, only patients with the appropriate ICD-9 code that was registered from the emergency room (department code: 02) and chronic colchicine users prior to the index date were excluded were included. Because cause-of-death information is not available in the NHIRD, CKD-related mortality in the present study refers to all-cause mortality among patients with established CKD rather than deaths directly attributable to renal failure. The date of death was ascertained from withdrawal from the National Health Insurance program, which has been validated as a reliable proxy for mortality in NHIRD-based studies.

The primary comparison was between colchicine users and nonusers among patients with heat-related injury, at a ratio of 1:4. Adjustment for potential confounders such as gout, pericarditis, autoimmune/inflammatory diseases. This study did not include a non-HRI control group; all analyses were restricted to patients with heat-related injury.

### 2.3. Definition of Colchicine Exposure Duration

Although the NHIRD does not capture actual medication adherence or precise treatment initiation and discontinuation dates, cumulative colchicine exposure was operationally approximated using prescription dispensing records. The total number of days supplied after the index HRI event was summed and categorized into three groups (<3 months, 3–12 months, and ≥1 year).

This duration-based classification reflects prescription records rather than confirmed drug intake and was used solely for exploratory analyses. Therefore, results from duration-stratified analyses should be interpreted cautiously and not as definitive dose–response relationships.

### 2.4. Propensity Score Model

The primary analytic cohort consisted exclusively of patients with heat-related injury, comparing colchicine users and nonusers using propensity score matching. A multivariate logistic regression model was applied to estimate the propensity score for patients who were administered colchicine within 7 days of HRI development. Subsequently, we estimated the outcome after PSM to control for confounding factors and ensure the comparability of colchicine users and nonusers. The PS model recorded potential confounding factors and covariates related to the disease outcome such as baseline medical comorbidities and medications used. Variables included in the propensity score model comprised demographic characteristics (age and sex), baseline comorbidities, and major clinical indications for colchicine use, including gout, pericarditis, and autoimmune or inflammatory diseases (including systemic lupus erythematosus, rheumatoid arthritis, psoriasis, multiple sclerosis, and type 1 diabetes mellitus.

### 2.5. Statistical Analysis

A cause-specific hazard model that was based on a big data analysis method model was employed. The 15-year adjusted cumulative hazard ratio (aHR) was estimated using the PS-matched cohort, in which each colchicine user in the exposed group was matched with four nonusers according to the year of HRI diagnosis. Furthermore, we estimated the cumulative risk of CKD or end-stage renal disease at different time points following colchicine initiation. All statistical analyses were performed using SAS software (version 9.4; SAS Institute Inc., Cary, NC, USA). Statistical tests were two-sided, and a *p*-value < 0.05 was considered statistically significant.

## 3. Results

A total of 6172 patients with heat-related injury (HRI) were initially identified as the source cohort. After applying the predefined exclusion criteria (*n* = 1211), 4961 eligible patients were included in the final analytic cohort. After propensity score matching, outcomes were compared exclusively between colchicine users and matched nonusers within the HRI cohort.

Demographic data of the matched cohort is presented in Table 1. The baseline characteristics were generally balanced after matching the patients in the NHIRD enrolled between 2000–2015. A total of 6172 patients with heat-related injury (HRI) who visited the emergency room or were hospitalized were identified to form the source HRI cohort. This included patients diagnosed with HRI before 2000, those aged <18 years or >100 years, those with a history of chronic kidney disease or renal replacement therapy, those with a previous history of kidney surgery, acute kidney injury, urinary system infection, genital organ anomalies, obstructive kidney disease, glomerulonephritis, or nephrotic syndrome, and those without racking or of an unknown sex, of whom 1211 were excluded.

**Figure 1:**
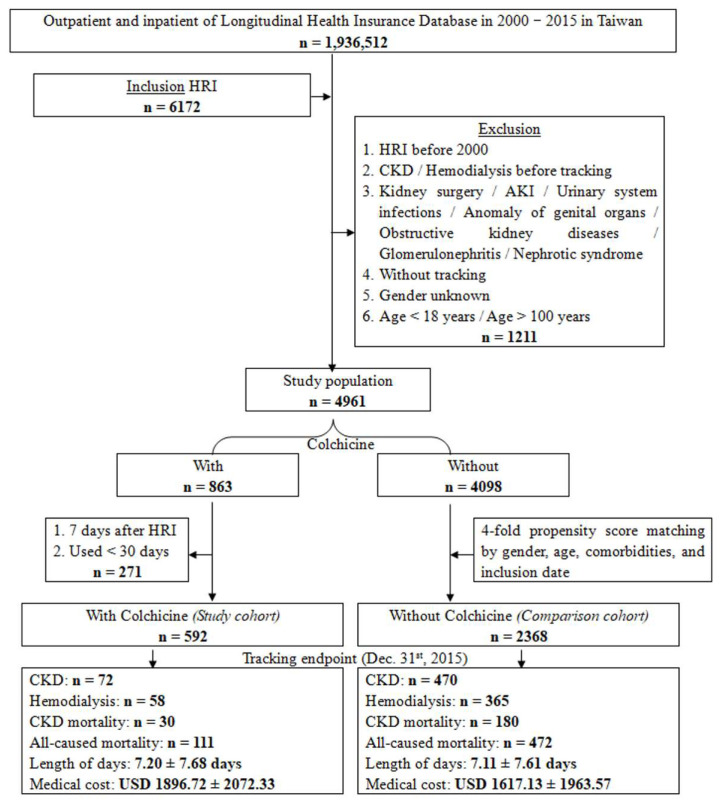
Flowchart of cohort selection and propensity score matching within the heat-related injury (HRI) cohort. Patients with HRI were classified as colchicine users or nonusers. CKD, chronic kidney disease; HRI, heat-related illness.

After applying the predefined inclusion and exclusion criteria, a total of 4961 patients with heat-related injury (HRI) were included in the final cohort. Among them, 592 patients received colchicine within 7 days after the index HRI event, and 2368 propensity score–matched patients without colchicine use served as the comparison group (Figure 1). The baseline demographic and clinical characteristics of the study population are presented in Table 1. The median follow-up duration was comparable between colchicine users and nonusers.

During the 15-year follow-up, we identified 72 (12.1%) CKD events, 58 (9.7%) hemodialysis events, 30 (5.0%) CKD-related deaths, and 111 all-cause deaths (18.7%) in the colchicine group. Furthermore, the length of stay and medical costs in the colchicine group were 7.20 ± 7.68 days and U.S. dollars (USD) 1896.72 ± 2072.33, respectively. In the comparison group, we identified 470 (19.8%) CKD events, 365 (15.4%) hemodialysis events, 180 (7.6%) CKD-related deaths, and 472 (19.9%) all-cause deaths. Furthermore, the length of stay and medical cost in the comparison group were 7.11 ± 7.61 days and USD 1617.13 ± 1963.57.

There were no differences in baseline demographic characteristics, sex, age, insurance premiums (in USD), major comorbidities, or clinical indications for colchicine use (including gout, pericarditis, and autoimmune/inflammatory diseases) and Charlson Comorbidity Index between the two groups after matching (Table 1).

**Figure 2:**
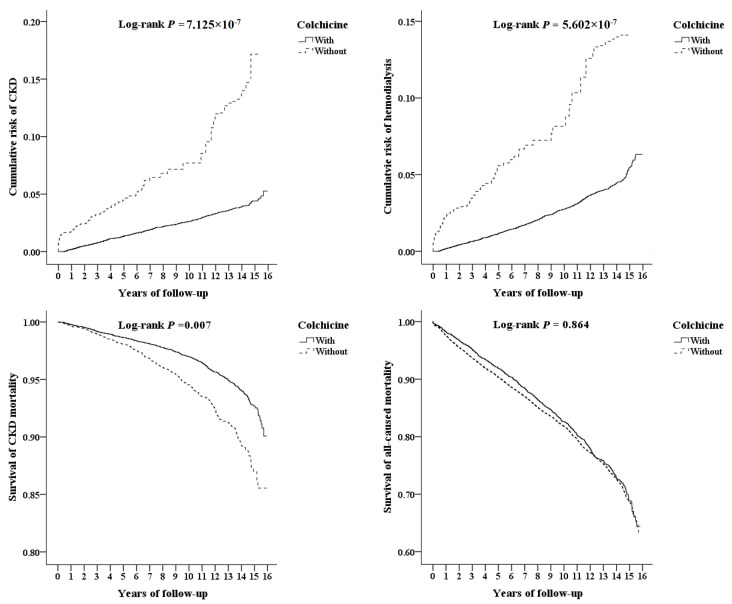
Kaplan–Meier curves of the outcomes. Chronic kidney disease, hemodialysis, chronic kidney disease-related mortality, and all-cause mortality were stratified by colchicine use using the log-rank test.

Kaplan–Meier curves demonstrated a significantly lower cumulative incidence of chronic kidney disease (CKD) and hemodialysis among colchicine users compared with propensity score–matched nonusers (Figure 2). Log-rank tests showed statistically significant differences between the two groups for CKD and hemodialysis outcomes. In contrast, no statistically significant difference was observed in all-cause mortality between colchicine users and nonusers during the follow-up period. A modest separation of curves was observed for CKD-related mortality; however, the difference was less pronounced than that observed for renal outcomes (Figure 2).

In adjusted Cox proportional hazards models, colchicine use was independently associated with a reduced risk of CKD progression (adjusted hazard ratio [aHR] = 0.675, *p* < 0.001) and hemodialysis (aHR = 0.592, *p* < 0.001) compared with nonuse (Table 2). Colchicine use was also associated with a lower risk of CKD-related mortality (aHR = 0.712, *p* < 0.001). However, no significant association was observed between colchicine use and all-cause mortality after multivariable adjustment (aHR = 0.739, *p* = 0.207). Increasing age, male sex, and major comorbidities—including diabetes mellitus, hypertension, septicemia, pneumonia, liver disease, and autoimmune diseases—were consistently associated with a higher risk of adverse renal and mortality outcomes (Table 2).

In analyses of medical utilization, colchicine use was not significantly associated with length of hospital stay after adjustment. However, colchicine users demonstrated higher medical costs, expressed in U.S. dollars (USD), compared with nonusers (adjusted relative risk for ln[medical cost] = 1.233, *p* < 0.001) (Table 3). Older age and male sex were also associated with increased medical costs, whereas length of stay was primarily influenced by demographic factors rather than colchicine use.

When colchicine exposure was stratified by cumulative prescription duration based on dispensing records, longer prescription duration was associated with more favorable renal outcomes. However, these findings should be interpreted as exploratory given the limitations of claims-based exposure ascertainment. (Table 4). Compared with nonusers, patients who received colchicine for ≥1 year exhibited the lowest risks of CKD (aHR = 0.670, *p* < 0.001), hemodialysis (aHR = 0.505, *p* < 0.001), and CKD-related mortality (aHR = 0.659, *p* < 0.001).

Shorter durations of colchicine use (<3 months and 3–12 months) were also associated with reduced risks of CKD and hemodialysis, although the magnitude of risk reduction was attenuated. No duration category of colchicine use was significantly associated with all-cause mortality.

## 4. Discussion

The purpose of care in advanced-stage CKD is to ameliorate CKD progression. However, improvements in CKD care and quality of life remain a tremendous challenge for policymakers. The kidney plays an important role in fluid and electrolyte regulation during heat stress and is a unique site for heat-induced diseases. The purpose of care in HRI is to ameliorate acute renal damage and CKD progression; however, this remains a tremendous challenge. In our study, we aimed to explore the effect of colchicine in patients with HRI.

After subgroup analysis and multivariate adjustment, we found that the colchicine treatment implemented by the multidisciplinary team was positively correlated with the outcome and reduced CKD risk, hemodialysis rate and CKD mortality. However, colchicine treatment was also associated with increased medical costs. Furthermore, the presence of multiple comorbidities in patients with HRI exerted a negative effect on progression to CKD, hemodialysis, CKD-mortality, and all-cause mortality. The benefit of colchicine therapy on the primary endpoint was more marked and statistically significant in patients diagnosed with HRI. The strategies of optimal management of patients with HRI include the implementation of nephroprotective measures. Prevention of the significant causes of CKD and reduction in mortality are the primary targets of these measures. Careful monitoring of the core body temperature, aggressive cooling of the patient, and early referral to a nephrologist are crucial and beneficial for an improved prognosis. Several immunomodulatory theories have been explored for HRI. In an animal study, elevated environmental temperatures resulted in increased blood flow toward the periphery to allow heat dissipation. This resulted in a decline in splanchnic blood flow, oxygen delivery, and nutrition delivery. The heat-induced gut dysfunction leads to oxidative stress, translocation of lumen contents, and release of proinflammatory mediators, which activates a systemic inflammatory response and causes dysregulation of the proinflammatory and anti-inflammatory balance [18].

The finding that colchicine use was associated with reduced CKD-related mortality but not with all-cause mortality warrants careful interpretation. CKD-related mortality represents a more disease-specific outcome that is directly linked to renal progression and its complications, whereas all-cause mortality encompasses a broad spectrum of competing risks, including cardiovascular disease, malignancy, infection, and trauma.

Given the advanced age and high comorbidity burden of patients with heat-related injury, it is plausible that non-renal causes of death may dilute any potential survival benefit attributable to renal protection alone. Moreover, the relatively limited number of cause-specific deaths may have reduced statistical power to detect differences in all-cause mortality. Taken together, these findings suggest that the potential benefit of colchicine may be more pronounced in renal-specific outcomes rather than overall survival.

Colchicine may mitigate these renal inflammatory processes through several mechanisms directly relevant to kidney protection. By inhibiting microtubule polymerization, colchicine suppresses neutrophil chemotaxis and adhesion, reduces inflammasome activation, and attenuates downstream cytokine release. These effects may help preserve renal microcirculation, limit inflammatory cell infiltration, and reduce progressive tubulointerstitial damage. Therefore, the observed association between colchicine use and reduced CKD progression in patients with heat-related injury may reflect modulation of inflammation-driven renal injury rather than nonspecific effects on systemic heat stress.

In our study, older adults demonstrated elevated risks of poorer outcomes than younger adults. The presence of DM, hypertension, heart failure, septicemia, pneumonia, and liver disease in patients is strongly associated with poorer outcomes [19,20]. Preexisting conditions such as cardiovascular disease, respiratory disease, renal failure, DM, and neurological disorders increase the susceptibility to heat stroke, thereby worsening the prognosis. Very young children and older adults are particularly vulnerable to heat stroke and its complications due to their impaired thermoregulatory mechanisms [21,22].

In our study, colchicine was associated with a statistically significant reduction in the risk of CKD and CKD-related mortality. However, there was no significant difference in the risk of all-cause mortality between the colchicine and control groups. Individuals with CKD have a higher risk of mortality than the general population. This may be attributable to cardiovascular complications such as heart failure or coronary artery disease, infection due to immunodeficiency, or metabolic disturbances such as acidosis, electrolyte imbalances, and anemia [23,24,25,26].

Several limitations should be acknowledged. First, due to the inherent constraints of claims-based data, the exact timing of colchicine initiation in relation to the heat-related injury could not be fully ascertained. Colchicine exposure may have occurred before HRI, been initiated during the acute phase, or reflected chronic use for underlying inflammatory conditions. Although we excluded patients with colchicine prescriptions prior to the index date in sensitivity analyses, misclassification of exposure timing cannot be completely ruled out. Second, despite the use of propensity score matching and adjustment for major comorbidities and colchicine-related indications (including gout, pericarditis, and systemic inflammatory diseases), residual confounding remains possible. Claims databases lack direct measures of disease severity, systemic inflammatory burden, lifestyle factors, and laboratory biomarkers, which may influence both colchicine prescription and renal outcomes.

Therefore, the observed associations should be interpreted cautiously as hypothesis-generating rather than causal. Finally, CKD may be undercoded/misclassified in claims data. Hence, larger trials are required for a better assessment of individual end points. Therefore, the safety profile of colchicine in this population could not be directly assessed. These limitations are common to large administrative databases and should be considered when interpreting the associations observed in the present study.

## 5. Conclusions

Our study findings suggest that colchicine may be beneficial in reducing the risk of developing CKD and CKD-related mortality in patients with HRI. However, further studies are required to validate our findings and explore the lack of a significant effect on all-cause mortality. Our findings also highlight the need for cautiously interpreting results from observational studies, especially those examining complex relationships between treatments and multiple health outcomes.

## Figures and Tables

**Table 1:** Characteristics of study at the baseline.

Colchicine	Total		With		Without		*p*
Variables	*n*	%	*n*	%	*n*	%	
Total	2960		592	20.00	2368	80.00	
Gender							0.999
Male	1920	64.86	384	64.86	1536	64.86	
Female	1040	35.14	208	35.14	832	35.14	
Age (years)	46.89 ± 19.06		46.72 ± 18.86		46.93 ± 19.11		0.811
Age group (yrs)							0.999
18–44	1260	42.57	252	42.57	1008	42.57	
45–64	1015	34.29	203	34.29	812	34.29	
≥65	685	23.14	137	23.14	548	23.14	
Insurance premium (NT$)							0.968
<18,000	2480	83.78	498	84.12	1982	83.70	
18,000–34,999	341	11.52	67	11.32	274	11.57	
≥35,000	139	4.70	27	4.56	112	4.73	
Diabetes mellitus							0.901
Without	2475	83.61	494	83.45	1981	83.66	
With	485	16.39	98	16.55	387	16.34	
Hypertension							0.724
Without	2405	81.25	478	80.74	1927	81.38	
With	555	18.75	114	19.26	441	18.62	
Heart failure							0.810
Without	2849	96.25	569	96.11	2280	96.28	
With	111	3.75	23	3.89	88	3.72	
Hyperlipidemia							0.839
Without	2627	88.75	524	88.51	2103	88.81	
With	333	11.25	68	11.49	265	11.19	
Hyperthyroidism							0.863
Without	2907	98.21	581	98.14	2326	98.23	
With	53	1.79	11	1.86	42	1.77	
Septicemia							0.532
Without	2857	96.52	569	96.11	2288	96.62	
With	103	3.48	23	3.89	80	3.38	
Pneumonia							0.629
Without	2797	94.49	557	94.09	2240	94.59	
With	163	5.51	35	5.91	128	5.41	
Liver disease							0.168
Without	2767	93.48	546	92.23	2221	93.79	
With	193	6.52	46	7.77	147	6.21	
CCI_R	0.81 ± 1.06		0.85 ± 1.11		0.80 ± 1.05		0.306

*p*: Chi-square/Fisher exact test on category variables and *t*-test on continue variables.

**Table 2:** Factors of outcomes by using Cox regression.

Outcomes	CKD		Hemodialysis		CKD-Mortality		All-Caused Mortality	
Variables	aHR	*p*	aHR	*p*	aHR	*p*	aHR	*p*
Colchicine								
Without	Reference		Reference		Reference		Reference	
With	0.670	<0.001	0.587	<0.001	0.701	<0.001	0.733	0.198
Gender								
Male	1.895	<0.001	1.435	<0.001	1.302	0.017	1.379	0.005
Female	Reference		Reference		Reference		Reference	
Age group (yrs)								
18–44	Reference		Reference		Reference		Reference	
45–64	1.598	<0.001	1.385	<0.001	1.576	<0.001	1.465	0.005
≥65	1.989	<0.001	1.650	<0.001	1.702	<0.001	1.606	<0.001
Diabetes mellitus								
Without	Reference		Reference		Reference		Reference	
With	2.562	<0.001	2.103	<0.001	2.304	<0.001	2.501	<0.001
Hypertension								
Without	Reference		Reference		Reference		Reference	
With	2.401	<0.001	2.264	<0.001	2.601	<0.001	2.331	<0.001
Heart failure								
Without	Reference		Reference		Reference		Reference	
With	1.065	0.373	1.466	0.076	1.115	0.191	2.065	<0.001
Hyperlipidemia								
Without	Reference		Reference		Reference		Reference	
With	1.103	0.201	1.650	0.001	1.504	0.023	1.230	0.111
Hyperthyroidism								
Without	Reference		Reference		Reference		Reference	
With	1.065	0.456	1.098	0.562	1.005	0.448	1.033	0.204
Septicemia								
Without	Reference		Reference		Reference		Reference	
With	1.498	<0.001	1.573	<0.001	1.356	0.041	2.065	<0.001
Pneumonia								
Without	Reference		Reference		Reference		Reference	
With	1.772	<0.001	1.897	<0.001	1.442	<0.001	2.382	<0.001
Liver disease								
Without	Reference		Reference		Reference		Reference	
With	1.505	<0.001	1.769	<0.001	1.980	<0.001	2.101	<0.001
CCI_R	1.202	<0.001	1.301	<0.001	1.222	<0.001	1.151	<0.001

aHR = Adjusted hazard ratio: Adjusted variables listed in the table.

**Table 3:** Factors of medical utilization by using linear regression.

Medical Utilization	ln (Length of Days)		ln (Medical Cost)	
Variables	aRR	*p*	aRR	*p*
Colchicine				
Without	Reference		Reference	
With	1.598	0.232	1.233	<0.001
Gender				
Male	1.264	0.046	1.101	0.188
Female	Reference		Reference	
Age group (yrs)				
18–44	Reference			
45–64	1.068	0.198	1.202	<0.001
≥65	1.198	0.084	1.356	<0.001
Diabetes mellitus				
Without	Reference		Reference	
With	2.506	<0.001	1.652	<0.001
Hypertension				
Without	Reference		Reference	
With	2.303	<0.001	1.440	<0.001
Heart failure				
Without	Reference		Reference	
With	1.860	<0.001	1.503	<0.001
Hyperlipidemia				
Without	Reference		Reference	
With	1.231	0.021	1.222	0.001
Hyperthyroidism				
Without	Reference		Reference	
With	1.065	0.311	1.165	0.398
Septicemia				
Without	Reference		Reference	
With	1.896	<0.001	1.774	<0.001
Pneumonia				
Without	Reference		Reference	
With	1.859	<0.001	1.565	<0.001
Liver disease				
Without	Reference		Reference	
With	1.706	<0.001	1.374	<0.001
CCI_R	1.303	<0.001	1.352	<0.001

aRR = Adjusted relative risk: Adjusted variables listed in the table.

**Table 4:** Factors of outcomes among different uses of colchicine by using Cox regression.

Outcomes	CKD		Hemodialysis		CKD-Mortality		All-Caused Mortality	
Colchicine	aHR	*p*	aHR	*p*	aHR	*p*	aHR	*p*
Without	Reference		Reference		Reference		Reference	
With	0.670	<0.001	0.587	<0.001	0.701	<0.001	0.733	0.198
<3 months	0.796	0.041	0.684	<0.001	0.864	0.189	0.829	0.243
≥3 months, <1 year	0.724	0.008	0.593	<0.001	0.735	0.039	0.704	0.162
≥1 year	0.663	<0.001	0.501	<0.001	0.653	<0.001	0.658	0.171

aHR = Adjusted hazard ratio: Adjusted variables listed in Table 3.

## Data Availability

The data presented in this study are available upon reasonable request from the Health and Welfare Data Science Center (HWDC), Ministry of Health and Welfare, Taiwan. Due to legal restrictions, the data cannot be made publicly available.
